# Double bypass for inoperable pancreatic malignancy at laparotomy: postoperative complications and long-term outcome

**DOI:** 10.1308/003588412X13373405386934

**Published:** 2012-05

**Authors:** F Ausania, AE Vallance, DM Manas, JM Prentis, CP Snowden, SA White, RM Charnley, JJ French, BC Jaques

**Affiliations:** Newcastle upon Tyne Hospitals NHS Foundation Trust,UK

**Keywords:** Pancreatic cancer, Palliative surgery, Postoperative complications

## Abstract

**INTRODUCTION:**

Between 4% and 13% of patients with operable pancreatic malignancy are found unresectable at the time of surgery. Double bypass is a good option for fit patients but it is associated with high risk of postoperative complications. The aim of this study was to identify pre-operatively which patients undergoing double bypass are at high risk of complications and to assess their long-term outcome.

**METHODS:**

Of the 576 patients undergoing pancreatic resections between 2006 and 2011, 50 patients who underwent a laparotomy for a planned pancreaticoduodenectomy had a double bypass procedure for inoperable disease. Demographic data, risk factors for postoperative complications and pre-operative anaesthetic assessment data including the Portsmouth Physiological and Operative Severity Score for the enUmeration of Mortality and morbidity (P-POSSUM) and cardiopulmonary exercise testing (CPET) were collected.

**RESULTS:**

Fifty patients (33 men and 17 women) were included in the study. The median patient age was 64 years (range: 39–79 years). The complication rate was 50% and the in-hospital mortality rate was 4%. The P-POSSUM physiology subscore and low anaerobic threshold at CPET were significantly associated with postoperative complications (*p*=0.005 and *p*=0.016 respectively) but they were unable to predict them. Overall long-term survival was significantly shorter in patients with postoperative complications (9 vs 18 months). Postoperative complications were independently associated with poorer long-term survival (*p*=0.003, odds ratio: 3.261).

**CONCLUSIONS:**

P-POSSUM and CPET are associated with postoperative complications but the possibility of using them for risk prediction requires further research. However, postoperative complications following double bypass have a significant impact on long-term survival and this type of surgery should therefore only be performed in specialised centres.

Even when state-of-the-art, high quality computed tomography (CT) is available, between 4% and 13% of pancreatic cancer patients are unresectable at surgical exploration.^1^ Biliary obstruction and gastric outlet obstruction are very common complications in patients with advanced pancreatic malignancy.^2^ Therapeutic options include surgical bypass (ie gastroenterostomy and hepaticojejunostomy), endoscopic retrograde cholangiopancreatography and percutaneous transhepatic cholangiography placement of stents.^3^

Retrospective studies of palliative surgical biliary bypass have reported mortality and morbidity rates of 3–16% and 28–48% respectively.^4,5^ There are no robust data on the role of double bypass surgery (ie gastric and biliary bypass surgery) versus endoscopic stenting in patients with biliary and duodenal/gastric outlet obstruction.^4^

Biliary stenting techniques are effective in the short term but may require further intervention and can be associated with recurrent attacks of cholangitis.^6,7^ Open surgical biliary bypass demonstrates low rates of recurrent jaundice (2–5%).^8^ For patients with gastric outlet obstruction, duodenal stents seem to be preferable in those with a short life expectancy and gastrojejunostomy may be preferable in patients with a more prolonged prognosis.^9^

In our unit, surgical bypass is preferred for patients who are fit for surgery, found to be inoperable at laparotomy and have a reasonable prognosis. Having been assessed for a pancreaticoduodenectomy, the majority of patients are presumed to be good candidates for a surgical bypass.

As recently demonstrated in our institution, complex medical co-morbidities predispose to postoperative complications after surgery and the majority of postoperative complications occur in a small group of high risk patients.^10^ The aim of this study was to identify the factors associated with postoperative complications in patients found inoperable at laparotomy and undergoing palliative double bypass for pancreatic malignancy using data collected from preoperative assessment. We also attempted to analyse the role of submaximal cardiopulmonary exercise testing (CPET), which produces a measurement of anaerobic threshold (AT), an independent predictor of increased postoperative complications.^10^

## Methods

### Patient population

The Freeman Hospital in Newcastle upon Tyne is a referral centre for pancreatic diseases in the north-east of England and the hepatopancreaticobiliary unit performs on average 120 pancreatic resections per year for malignancy. All patients who underwent a double bypass procedure between January 2006 and July 2011 at the hepatopancreatobiliary unit were identified from a prospectively held database.

Only patients with pre-operatively resectable pancreatic malignancy or those with borderline resectability were included in the study. Patients without histological evidence of pancreatic malignancy were excluded. Endoscopic ultrasonography was performed only when tissue diagnosis was not available or when better assessment of vascular invasion was needed.

Contraindications for resectability were: superior mesenteric artery, hepatic artery and coeliac artery involvement; superior mesenteric vein involvement below the first mesenteric branches; liver metastases; peritoneal metastases; and inferior vena cava involvement.

In all cases the double bypass surgery consisted of double jejunal loop reconstruction with an end-to-side hepaticojejunostomy on a Roux-en-Y with a loop gastroenterostomy.

Patients without up-to-date (within four weeks of surgery) CT were excluded from the study as this clearly relates to outcome of patients found to be unresectable at surgery. None of these patients received pre-operative chemotherapy or radiotherapy. Patients with neuroendocrine tumours were also excluded from the study as they have a less aggressive disease.

All patients were assessed routinely for pancreaticoduodenectomy. The American Society of Anesthesiologists (ASA) physical status classification system was adopted (grade 1/2 = normal healthy or mild systemic disease; grade 3 = severe systemic disease; grade 4/5 = severe systemic disease that is a constant threat to life or moribund). The Portsmouth Physiological and Operative Severity Score for the enUmeration of Mortality and morbidity (P-POSSUM) physiology subscore (age, cardiac and respiratory status, electrocardiography report, systolic blood pressure, pulse rate, haemoglobin, white cell count, urea, sodium, potassium, Glasgow coma scale) was also calculated as it has been validated previously as a very good predictor of morbidity in pancreatic surgery.^11,12^

Patients were also selected for CPET by virtue of a low subjective functional capacity. This was defined by a metabolic equivalent score of ≤7, according to a simple activity-based clinical history. In our institution, we have demonstrated previously (unpublished data) that patients with a simple metabolic equivalent score of ≤7 do not predictably have major complications postoperatively. CPET is the analysis of both cardiac and pulmonary data during exercise. It measures both O_2_ consumption and CO_2_ production, and can identify the point at which CO_2_ levels start to increase at the airway. This correlates to the lactate, or AT where energy systems begin to change over.

### Outcome measures

Postoperative complications were recorded according to the postoperative morbidity survey.^13^ Secondary outcomes included intensive care unit stay, hospital stay, in-hospital mortality rates, delay or cancellation of palliative chemotherapy due to complications and overall survival. Patients were reviewed routinely in outpatient clinics every three months.

All data (including demographic details, medical history, intra-operative details, morbidity and mortality, palliative chemotherapy and overall survival) were collected from a prospectively held database. Patients with in-hospital mortality were excluded from the long-term survival analysis.

### Statistical analysis

T-test (continuous, normal distribution), Mann–Whitney (continuous, non-normal distribution) and chi-square (categorical) analyses were used to compare demographic and clinical variables between groups. A two-tailed *p*-value of <0.05 was considered significant. The receiver operating characteristic (ROC) curve was used to determine optimal values (upper left corner), area under the curve (AUC), sensitivity and specificity. Survival was calculated by the Kaplan–Meier method and Cox proportional hazards regression. All analyses were performed using SPSS® 19.0 (SPSS, Chicago, IL, US). A *p*-value of <0.05 was considered significant.

## Results

Out of 576 patients who had an operation for pancreatic malignancy between January 2006 and July 2011, 57 patients (10%) underwent palliative double bypass surgery. All patients had a pre-operative biliary stent. (Usually, a plastic stent was used; four patients had a short metal stent.) No patients had pre-operative gastric outlet obstruction. Four patients with a diagnosis of neuroendocrine malignancy and three patients who were not considered for a pancreaticoduodenectomy at the time of the bypass were excluded. Three patients who did not have up-to-date CT underwent a palliative gastroenterostomy.

Fifty patients were considered in the study. The population data are shown in Table 1. The median age was 64 years (range: 39–79 years), and 33 male and 17 female patients were evaluated. The most common indication for surgery was pancreatic ductal adenocarcinoma (*n*=35, 70%). Postoperative complications occurred in 50% of patients (*n*=25). In-hospital mortality was 4% (*n*=2). The median hospital stay postoperatively was 12.6 days (range: 5–39 days). No patients were lost at follow-up. At the time of analysis, 16 patients (32%) were still alive. The median follow-up duration was 10 months (range: 4–33 months). Reasons for inoperability are also shown in Table 1.

**Table 1:** Population baseline characteristics

Median age in years (range)	64 (39–79)
Sex	
> Male	33 (66%)
> Female	17 (34%)
Median body mass index in kg/m^2^ (range)	25.5 (18–42)
Indication to surgery	
> Ductal carcinoma	35 (70%)
> Ampullary carcinoma	4 (8%)
> Duodenal carcinoma	4 (8%)
> Cholangiocarcinoma	1 (2%)
> Undifferentiated adenocarcinoma	6 (12%)
Reason for palliative surgery	
> SMA, CA, HA, SMV involvement	28 (56%)
> Liver metastases	15 (30%)
> Peritoneal metastases	4 (8%)
> More than one of the above reasons	3 (6%)
Postoperative complications	25 (50%)
More than 1 complication	9 (18%)
Type of complication (POMS)*	
> Cardiovascular	2 (4%)
> Pulmonary	6 (12%)
> Renal	3 (6%)
> Gastrointestinal (including GOO)	5 (10%)
> Anastomotic leak (biliary, gastric or enteric)	6 (12%)
> Others (wound infection, uncontrolled diabetes etc)	12 (24%)
Median length of hospital stay in days (range)	12.6 (5–39)
Relaparotomy	3 (6%)
In-hospital mortality	2 (4%)

SMA = superior mesenteric artery; CA = coeliac artery; HA = hepatic artery; SMV = superior mesenteric vein; GOO = gastric outlet obstruction

* POMS (postoperative morbidity survey): anastomotic leak is shown as a separate complication

Primary and secondary outcomes are shown in Table 2. Twenty of the fifty patients had pre-operative CPET. P-POSSUM physiology subscore and AT are significantly associated with postoperative complications (*p*=0.005 and *p*=0.016 respectively).

**Table 2:** Outcome variables comparing patients with and without complications

	No complications (*n*=25)	1 or more complications (*n*=25)	P-value
Median age in years (range)	64.6 (45–79)	64.6 (39–76)	1.0
Sex			0.551
> Male	15	18	
> Female	10	7	
Indication for surgery			0.731
> Ductal carcinoma	18	17	
> Ampullary carcinoma	1	3	
> Duodenal carcinoma	2	2	
> Cholangiocarcinoma	1	0	
> Undifferentiated adenocarcinoma	3	3	
Reason for palliative surgery			0.681
> SMA, CA, HA, SMV involvement	16	12	
> Liver metastases	6	9	
> Peritoneal metastases	2	2	
> More than one of the above reasons	1	2	
Median body mass index in kg/m^2^ (range)	25.5 (21–42)	25.4 (18–39)	0.954
Median blood loss at surgery in ml (range)	733 (100–2,310)	837 (250–2,500)	0.491
Operating time in hours (range)	4.0 (1.5–6)	4.2 (2–7)	0.553
Biliary stent at surgery	19	19	1.0
Bilirubin level at surgery in μmol/l (range)	36.0 (3–100)	63.6 (6–208)	0.064
Diabetes	7	7	1.0
Cardiac history	6	6	1.0
ASA grade			0.053
> 2	20	12	
> 3	5	12	
> 4	0	1	
Median P-POSSUM physiology score (range)	16.5 (12–20)	18.7 (15–26)	**0.005**
Median anaerobic threshold in ml/kg/min (range)*	14.1 (10.3–16.9)	11.3 (6.2–15.4)	**0.016**
Chemotherapy following surgery	16	12	0.693
Chemotherapy not given as prolonged hospital stay	0	3	0.235

SMA = superior mesenteric artery; CA = coeliac artery; HA = hepatic artery; SMV = superior mesenteric vein; ASA = American Society of Anesthesiologists; P-POSSUM = Portsmouth Physiological and Operative Severity Score for the enUmeration of Mortality and morbidity

* 20 patients only

The median P-POSSUM physiology subscore in the complications group was 18.7. The ROC curve showed an optimal P-POSSUM physiology subscore at 16.5 with a fair degree of accuracy (AUC 70%, *p*=0.013), and a sensitivity and specificity of 72% and 56% respectively. The ROC curve was not performed on AT due to the small number of patients.

**Figure 1:**
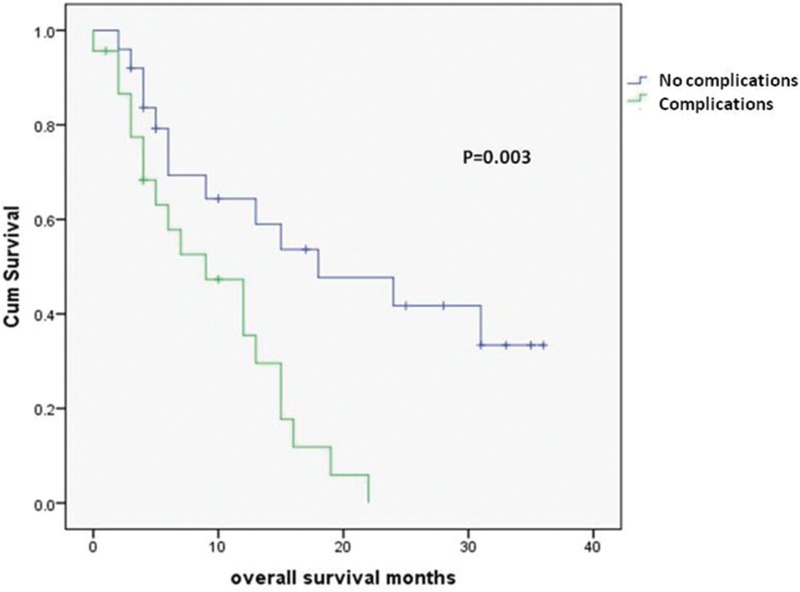
Survival for patients with and without postoperative complications

The median overall survival for all patients was 14.6 months. Patients with postoperative complications had a significantly shorter median survival than those without complications (9 vs 18 months, *p*=0.003) (Fig 1).

At univariate analysis, age at surgery, sex, tumour histology and reason of inoperability were not significantly associated with long-term survival. At Cox regression analysis, neither P-POSSUM or AT were associated with overall long-term survival (*p*=0.274 and *p*=0.498 respectively). At multivariate analysis, postoperative complications were associated independently with poorer long-term survival (*p*=0.003, odds ratio [OR]: 3.261, 95% confidence interval [CI]: 1.492–7.129). Palliative chemotherapy was also significantly associated with survival (*p*=0.028, OR: 0.446, 95% CI: 0.215–0.924). No multicollinearity was evidenced between the two variables.

## Discussion

There is currently no strong evidence on how to manage patients with inoperable disease at laparotomy. Most of the literature has focused on comparing surgical bypass with biliary stents in biliary obstruction. Three randomised studies that compared surgical bypass with plastic biliary stenting found no difference in the rates of technical or therapeutic success.^14–16^ However, although the relative risk of complications was lower in the stenting group, there was a highly significant reduction in recurrent biliary obstruction at four months in the surgical group.

Laparoscopic biliary bypass has also been developed as a minimally invasive approach^17^ but there is no randomised evidence to show superiority when comparing open and laparoscopic biliary bypass surgery.^3^ With regard to gastric outlet obstruction, which occurs in 10–20% of pancreatic cancer patients,^18^ laparoscopic gastroenterostomy has been introduced as a minimally invasive approach to surgical bypass and there is some evidence of a quicker recovery rate^19^ although duodenal stents may be preferable in patients with a short life expectancy.^18^

One of the questions we tried to answer was whether it is possible to identify those patients with a high risk of complications and to perhaps consider avoiding a surgical bypass at the time of laparotomy. We demonstrated that P-POSSUM physiology subscore and AT (albeit with a small sample size) are significantly associated with postoperative complications but we failed to validate these tests for risk prediction.

In fact, one of the problems we encountered was that all our patients were candidates for elective pancreaticoduodenectomy and therefore relatively fit. The median P-POSSUM physiology subscore in the complications group was low, making difficult to use this test to predict complications as evidenced by the ROC curve. The same applied to AT (11.3ml/kg/min in the complications group), which was higher when compared with our previous analysis, where an AT of <10.1 was used to predict complications.^20^ We believe larger studies are needed to test P-POSSUM and AT as predictors of complications.

There is very little literature looking into risk prediction in these patients. In 2009 de Castro *et al* published a study showing that POSSUM overpredicts morbidity and predicts overall long-term survival in patients with unresectable pancreatic cancer.^21^ In this study, surprisingly, 32% of the patients were found unresectable at laparotomy, possibly because of historical data, in contrast with the literature reporting an unresectability rate at laparotomy of 4–13%.

POSSUM includes the same variables as P-POSSUM but a different formula is used to predict the risk of death^11^ and it consists of 12 physiological factors (age, cardiac status, respiratory status, electrocardiography report, systolic blood pressure, pulse rate, Glasgow coma scale, haemoglobin, white cell count, urea, sodium, potassium) and 6 operative parameters (operative complexity, multiple procedures, blood loss, peritoneal contamination, extent of malignant spread, elective or emergency surgery). Obviously, these last parameters are very difficult to assess before surgery and therefore the role of POSSUM in this study is very limited.

An interesting finding of our study was that complications have a significant impact on long-term survival. In 2012 Kamphues *et al* published the long-term outcomes of 428 patients who underwent resection of pancreatic head cancer.^22^ The median survival was 15.5 months with a postoperative complication rate of 32.7%. The occurrence of severe postoperative complications was associated with a significantly shortened survival compared with patients without complications (16.5 vs 12.4 months; *p*=0.002) and was identified as an independent prognostic factor (*p*=0.002). Similar results have been shown in almost every type of gastrointestinal malignancy.^23^

As other studies have indicated, long-term survival after surgery may be affected by the degree of systemic immune response to the infection or surgical trauma shifted toward a T_h_2-type lymphocyte pattern.^24–26^ IL-10, one of the T_h_2 cytokines, downregulates tumour specific immune responses by directly suppressing IFNc and IL-12 production, thereby reducing major histocompatibility complex expression on the surface of tumour cells and inhibited tumour antigen presentation by antigen presenting cells.^27–29^ In line with these studies, we have demonstrated that severe postoperative complications have a strong impact on the long-term survival of patients with inoperable pancreatic head cancer and, as a result, these patients should be treated only in specialised centres.

## Conclusions

Postoperative complications are associated independently with poorer long-term outcome in patients with pancreatic malignancy found unresectable at laparotomy. This type of surgery should therefore only be performed in specialised centres. A high P-POSSUM physiology subscore and a low cardiopulmonary reserve are pre-operative parameters associated with postoperative complications but further studies are needed to clarify their value as risk predictors. This would allow a better selection of patients who really benefit from palliative surgery at the time of laparotomy.

## References

[1] Pisters PW, Lee JE, Vauthey JN*et al* Laparoscopy in the staging of pancreatic cancer. Br J Surg 2001; 88: 325–337 11260096 10.1046/j.1365-2168.2001.01695.x

[2] Watanabe I, Sasaki S, Konishi M*et al* Onset symptoms and tumor locations as prognostic factors of pancreatic cancer. Pancreas 2004; 28: 160–165 15028948 10.1097/00006676-200403000-00007

[3] Huggett MT, Ghaneh P, Pereira SP. Drainage and bypass procedures for palliation of malignant diseases of the upper gastrointestinal tract. Clin Oncol 2010; 22: 755–763 10.1016/j.clon.2010.08.001 PMC2978505 20805023

[4] Mann CD, Thomasset SC, Johnson NA*et al* Combined biliary and gastric bypass procedures as effective palliation for unresectable malignant disease. ANZ J Surg 2009; 79: 471–475 19566872 10.1111/j.1445-2197.2008.04798.x

[5] Kuhlmann KF, van Poll D, de Castro SM*et al* Initial and long-term outcome after palliative surgical drainage of 269 patients with malignant biliary obstruction. Eur J Surg Oncol 2007; 33: 757–762 17215099 10.1016/j.ejso.2006.11.014

[6] Distler M, Kersting S, Rückert F*et al* Palliative treatment of obstructive jaundice in patients with carcinoma of the pancreatic head or distal biliary tree. Endoscopic stent placement vs hepaticojejunostomy. JOP 2010; 11: 568–574 21068488

[7] Artifon EL, Sakai P, Cunha JE*et al* Surgery or endoscopy for palliation of biliary obstruction due to metastatic pancreatic cancer. Am J Gastroenterol 2006; 101: 2,031–2,037 16968509 10.1111/j.1572-0241.2006.00764.x

[8] Moss AC, Morris E, Leyden J, MacMathuna P. Malignant distal biliary obstruction: a systematic review and meta-analysis of endoscopic and surgical bypass results. Cancer Treat Rev 2007; 33: 213–221 17157990 10.1016/j.ctrv.2006.10.006

[9] Jeurnink SM, van Eijck CH, Steyerberg EW*et al* Stent versus gastrojejunostomy for the palliation of gastric outlet obstruction: a systematic review. BMC Gastroenterol 2007; 7: 18 17559659 10.1186/1471-230X-7-18 PMC1904222

[10] Snowden CP, Prentis JM, Anderson HL*et al* Submaximal cardiopulmonary exercise testing predicts complications and hospital length of stay in patients undergoing major elective surgery. Ann Surg 2010; 251: 535–541 20134313 10.1097/SLA.0b013e3181cf811d

[11] Prytherch DR, Whiteley MS, Higgins B*et al* POSSUM and Portsmouth POSSUM for predicting mortality. Physiological and Operative Severity Score for the enUmeration of Mortality and morbidity. Br J Surg 1998; 85: 1,217–1,220 10.1046/j.1365-2168.1998.00840.x 9752863

[12] Tamijmarane A, Bhati CS, Mirza DF*et al* Application of Portsmouth modification of physiological and operative severity scoring system for enumeration of morbidity and mortality (P-POSSUM) in pancreatic surgery. World J Surg Oncol 2008; 6: 39 18400108 10.1186/1477-7819-6-39 PMC2346467

[13] Bennett-Guerrero E, Welsby I, Dunn TJ*et al* The use of a postoperative morbidity survey to evaluate patients with prolonged hospitalization after routine, moderate-risk, elective surgery. Anesth Analg 1999; 89: 514–519 10439777 10.1097/00000539-199908000-00050

[14] Smith AC, Dowsett JF, Russell RC*et al* Randomised trial of endoscopic stenting versus surgical bypass in malignant low bile duct obstruction. Lancet 1994; 344: 1,655–1,660 7996958 10.1016/s0140-6736(94)90455-3

[15] Andersen JR, Sørensen SM, Kruse A*et al* Randomised trial of endoscopic endoprosthesis versus operative bypass in malignant obstructive jaundice. Gut 1989; 30: 1,132–1,135 10.1136/gut.30.8.1132 PMC1434171 2475392

[16] Shepherd HA, Royle G, Ross AP*et al* Endoscopic biliary endoprosthesis in the palliation of malignant obstruction of the distal common bile duct: a randomized trial. Br J Surg 1988; 75: 1,166–1,168 10.1002/bjs.1800751207 2466520

[17] Hamade AM, Al-Bahrani AZ, Owera AM*et al* Therapeutic, prophylactic, and preresection applications of laparoscopic gastric and biliary bypass for patients with periampullary malignancy. Surg Endosc 2005; 19: 1,333–1,340 16021372 10.1007/s00464-004-2282-4

[18] Jeurnink SM, Steyerberg EW, van Hooft JE*et al* Surgical gastrojejunostomy or endoscopic stent placement for the palliation of malignant gastric outlet obstruction (SUSTENT study): a multicenter randomized trial. Gastrointest Endosc 2010; 71: 490–499 20003966 10.1016/j.gie.2009.09.042

[19] Mehta S, Hindmarsh A, Cheong E*et al* Prospective randomized trial of laparoscopic gastrojejunostomy versus duodenal stenting for malignant gastric outflow obstruction. Surg Endosc 2006; 20: 239–242 16362479 10.1007/s00464-005-0130-9

[20] Ausania F, Snowden CP, Prentis JM*et al* Effects of low cardiopulmonary reserve on pancreatic leak following pancreaticoduodenectomy. Br J Surg 2012; 99: 1,290–1,294 10.1002/bjs.8859 22828960

[21] de Castro SM, Houwert JT, Lagard SM*et al* POSSUM predicts survival in patients with unresectable pancreatic cancer. Dig Surg 2009; 26: 75–79 19169034 10.1159/000194982

[22] Kamphues C, Bova R, Schricke D*et al* Postoperative complications deteriorate long-term outcome in pancreatic cancer patients. Ann Surg Oncol 2012; 19: 856–863 21879265 10.1245/s10434-011-2041-4

[23] Okamura Y, Takeda S, Fujii T*et al* Prognostic significance of postoperative complications after hepatectomy for hepatocellular carcinoma. J Surg Oncol 2011; 104: 814–821 21713775 10.1002/jso.21977

[24] Aosasa S, Ono S, Mochizuki H*et al* Activation of monocytes and endothelial cells depends on the severity of surgical stress. World J Surg 2000; 24: 10–16 10594196 10.1007/s002689910003

[25] Mokart D, Capo C, Blache JL*et al* Early postoperative compensatory anti-inflammatory response syndrome is associated with septic complications after major surgical trauma in patients with cancer. Br J Surg 2002; 89: 1,450–1,456 10.1046/j.1365-2168.2002.02218.x 12390391

[26] Tsujimoto H, Ono S, Majima T*et al* Differential toll-like receptor expression after ex vivo lipopolysaccharide exposure in patients with sepsis and following surgical stress. Clin Immunol 2006; 119: 180–187 16517212 10.1016/j.clim.2006.01.004

[27] Clerici M, Shearer GM, Clerici E. Cytokine dysregulation in invasive cervical carcinoma and other human neoplasias: time to consider the TH1/TH2 paradigm. J Natl Cancer Inst 1998; 90: 261–263 9486806 10.1093/jnci/90.4.261

[28] Beissert S, Hosoi J, Grabbe S*et al* IL-10 inhibits tumor antigen presentation by epidermal antigen-presenting cells. J Immunol 1995; 154: 1,280–1,286 7822797

[29] Mynster T, Christensen IJ, Moesgaard F, Nielsen HJ. Effects of the combination of blood transfusion and postoperative infectious complications on prognosis after surgery for colorectal cancer. Br J Surg 2000; 87: 1,553–1,562 10.1046/j.1365-2168.2000.01570.x 11091245

